# Congenital Hepatic Fibrosis in Children and Adults: Clinical Manifestations, Management, and Outcome—Case Series and Literature Review

**DOI:** 10.1155/2020/8284274

**Published:** 2020-04-21

**Authors:** Beidi Zhu, Zunguo Du, Zhengxin Wang, Yang Li, Jiming Zhang, Haoxiang Zhu

**Affiliations:** ^1^Department of Infectious Diseases, Huashan Hospital, Fudan University, 12 Middle Wulumuqi Road, Shanghai 200040, China; ^2^Department of Pathology, Huashan Hospital, Fudan University, 12 Middle Wulumuqi Road, Shanghai 200040, China; ^3^Department of General Surgery, Huashan Hospital, Fudan University, 12 Middle Wulumuqi Road, Shanghai 200040, China

## Abstract

**Background:**

Congenital hepatic fibrosis is a hereditary fibropolycystic disease caused by ductal plate malformation. It is characterized by portal hypertension, but the manifestations, management, and outcome vary in children and adults. To raise awareness of medical staff, we have comprehensively compared the clinical features of congenital hepatic fibrosis between children and adults.

**Methods:**

We retrospectively enrolled all patients diagnosed with congenital hepatic fibrosis at the Huashan Hospital from August 2015 to August 2017 and analyzed their familial, clinical, laboratory, imaging, treatment, and follow-up data in detail. In addition, we reviewed cases with congenital hepatic fibrosis reported in the past 20 years in China and analyzed them according to the patients' age.

**Results:**

A total of eight patients were diagnosed with congenital hepatic fibrosis in the study, including four children and four adults. The onset age of the children, who suffered from severe complications of portal hypertension and needed liver transplantation, ranged from 1 to 15 years old. The disorder developed in adults aged 26 to 60 years old. Three adults complained of recurrent abnormal liver function at the onset of illness, and they mainly received conservative treatments. The literature review included 30 children and 33 adults. In comparison, hepatomegaly was more common in children than in adults (57% *vs*. 21%, *p* = 0.004). Malformation of kidneys and bile duct abnormalities were common, and multisystem involvement included eyes, other digestive organs, and genital and central nervous systems.

**Conclusions:**

Serious complications of portal hypertension developed in children requiring liver transplantation, while adults often had mild-to-moderate liver injuries upon onset. Adults with CHF varied a lot in clinical manifestations. Multiorgan involvement and unusual course are helpful to make a diagnosis. Timely histological assessment by liver biopsy and multidisciplinary cooperation are crucial for definitive diagnosis and early intervention.

## 1. Introduction

Congenital hepatic fibrosis (CHF) is a rare developmental disorder pathologically based on ductal plate malformation (DPM), namely, ciliopathy or fibrocystic liver disease. It is characterized by hepatosplenomegaly and portal hypertension. The prevalence of the disease is 1/10000–20000 [1, 2]. CHF rarely presents as a single entity but is often concomitant with a wide range of disorders caused by various gene mutations like autosomal recessive polycystic kidney disease (ARPKD) and Caroli syndrome. Nowadays, pediatric-onset liver disorders are increasingly common in adult hepatology practices due to latent pathogenesis and more awareness about congenital disease [3]. The severity and prognosis of this disease differ according to onset ages, and the therapeutic regimens vary accordingly. Therefore, we retrospectively analyzed and compared the clinical features of four children and four adults definitively diagnosed with CHF by liver histopathology. Furthermore, we reviewed and analyzed 63 cases with CHF reported in the past 20 years in China according to onset age.

## 2. Materials and Methods

We retrospectively enrolled all of the CHF cases that were diagnosed pathologically at the Huashan Hospital in Shanghai, China, from August 2015 to August 2017, and then collected the clinical data, including demographic information, family history, clinical manifestations, laboratory indexes, imaging findings, treatment, and follow-up. Then, we summarized and compared the clinical features of CHF according to onset age.

Liver histopathology is the gold standard for CHF diagnosis, which was performed by postliver transplant biopsy or ultrasound-guided percutaneous biopsy using a 16 g Tru-Cut needle (USA, Argon). All CHF cases in our study met the pathological characteristics of CHF, including the following: (1) defective remodeling of the ductal plate manifesting as abnormally shaped small bile ducts in the portal area and the cubic or columnar bile duct epithelia form elongated or cystic cavities, (2) dense fibrous septa of different widths separate liver parenchyma into hepatic islands containing normal vasculature and portal-portal bridging fibrosis and intact hepatic lobule, and (3) potentially abnormal changes to the intrahepatic portal vein branches. Cystic expansions of the intrahepatic bile ducts (microscopic and medium-sized bile ducts) are characteristics of Caroli syndrome, which was considered to be a different stage of the same disease as CHF in which microscopic bile ducts are involved [4]. Genetic testing was performed for 2 patients, in whom the candidate gene selected on the basis of clinical features and family history was tested by Sanger sequencing (*ABCB4* gene for Patient 4 and *PKHD1* gene for Patient 5).

In addition, we searched Chinese databases (China National Knowledge Internet, WanFang Data, and SinoMed) and reviewed 63 cases with CHF reported in the past 20 years in China. Comparisons between the clinical features of the children and adults were made through the chi-squared and Fisher's exact tests using IBM SPSS Statistics 22 (IBM).

## 3. Results

### 3.1. Study Population

A total of four pediatric patients and four adult-aged patients were enrolled. All of the adults and the oldest child were diagnosed with CHF by ultrasound-guided percutaneous liver biopsy, and the other children's diagnoses were ultimately confirmed after liver transplantation owing to liver decompensation. All four pediatric patients were admitted to the department of liver surgery, including a 13-month-old boy (P1), a 4-year-old girl (P2), a 15-year-old boy (P3), and a 9-year-old girl (P4). All four adult patients were hospitalized in the internal medicine department, including three female patients aged 26, 26, and 60 years (P5, P7, and P8, respectively) and a 26-year-old male patient (P6).

### 3.2. Case Reports

Patients 1-4 were all pediatric patients. Patient 1 developed cholangitis and hepatosplenomegaly at the 7^th^ month after Kasai surgery for congenital biliary atresia (CBA), and then, he accepted the operation of piggyback liver transplantation and cholangioenteric anastomosis. Patient 2 suffered from severe liver injury and repeated hematemesis caused by gastric fundus varicosis. So she underwent living donor liver transplantation (LDLT) for liver decompensation. Patient 3 had a history of polycystic liver and kidneys. Recurrent hematemesis and melena happened for 8 years, and eventually, he was diagnosed as Caroli syndrome based on radiology and histology. In Patients 1-3, posttransplant pathology confirmed the diagnosis of CHF. Patient 4 presented with repeated melena, bloating, and itching for two months and then developed severe anemia, cholestasis, and ascites. *ABCB4* mutation indicated the probability of progressive familial intrahepatic cholestasis-type 3 (PFIC3). Liver biopsy histologically conformed to features of CHF. This patient has been awaiting liver transplantation until now.

Patients 5-8 were adult patients. Patient 5 had of a history of chronic hepatitis B (CHB) and polycystic kidneys. This patient rapidly developed ascites, hypoalbuminemia, and oliguria under regular antiviral treatment. Thus, the previous pathological section was assessed again and considered to be CHB overlapping CHF. Patient 6, Patient 7, and Patient 8 presented with mildly-to-moderately elevated transaminase and bilirubin levels and progressive spleen enlargement. Patient 6 was initially diagnosed with intrahepatic cholestasis of pregnancy (ICP), yet there was no improvement after parturition and taking ursodeoxycholic acid. So liver puncture was performed in these three adults, through which they were diagnosed with CHF and at a long-term follow-up.

### 3.3. Clinical Manifestations

The eight cases presented with different symptoms and durations (Table 1). The youngest child (P1) previously suffered from obstructive jaundice caused by CBA and underwent Kasai operation at 3 months old. He was hospitalized again because of a high postoperatively fever, hepatosplenomegaly, and cholestatic liver injury. The other three pediatric cases (P2, P3, and P4) all suffered from repeated hematemesis or melena, suggestive of upper gastrointestinal bleeding. Unlike the children, the majority of adults (P6, P7, and P8) were hospitalized due to abnormal liver function. Notably, Patient 5 had a 20-year history of polycystic kidney and untreated CHB with pathological grade G3S4 and then underwent splenectomy for severe hypersplenism and esophageal varices. She mainly presented with abdominal distension, edema, oliguria, and anemia. Only one patient (P3) had a suspicious family history, namely, an older sister who developed cirrhosis at 29 years old and a younger sister who underwent a splenectomy for hypersplenism at 27 years old.

### 3.4. Laboratory Data

Laboratory data are presented in Table 1 in detail. Although more adults were admitted for repeated abnormal liver function, their liver enzymes were no more than three times the upper limit of normal (ULN). Transaminase levels were significantly higher in children (ALT, 95% CI: 2.06–5.94 ULN; AST, 95% CI: 2.34–5.95 ULN) than in adults (ALT, 95% CI: 0.59–1.21 ULN, *p* = 0.014; AST, 95% CI: 0.75–1.31 ULN, *p* = 0.012). There is no significant difference for ALP, GGT, bilirubin, INR, or albumin between children and adults. There was no correlation between the onset age and the level of albumin (Pearson *R* = 0.594, *p* = 0.121) or the coagulation function (Pearson *R* = 0.688, *p* = 0.06), which was mostly in the normal range. All children had anemia, and the most severe case (P3) had an 8-year history of recurrent hematemesis. Indicators of iron metabolism showed iron deficiency anemia in two cases (P1, P4). Leucocyte levels decreased moderately in two children (P2, P4). One adult (P5) had severe anemia because of chronic kidney disease (CKD) caused by polycystic kidneys. In this patient, the creatinine level was 189 *μ*mol/L and the glomerular filtration rate was estimated around 31 mL/(min·1.73m^2^) by the CKD epidemiology collaboration (CKD-EPI) equation. In the other three adults, only platelet levels decreased. Except that hepatitis B surface antigen (HBsAg), hepatitis B e antibody (HBeAb), and hepatitis B core antibody (HBcAb) were positive in Patient 5, the presence of viral hepatitis, hepatic parasitosis, hematopathy, and autoimmune and metabolic liver diseases was excluded through laboratory tests.

### 3.5. Imaging Findings

**Figure 1:**
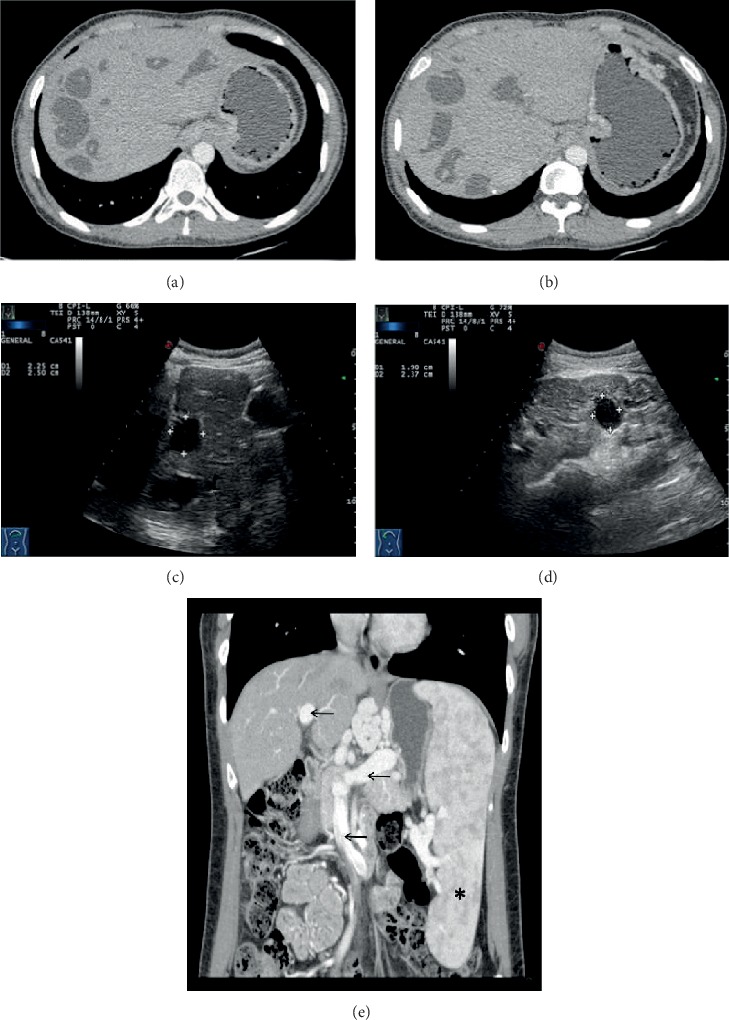
Portal vein CT venography- (CTV-) enhanced scan (a, b) and abdominal ultrasound (c, d) of P5 suggested dilation of the intrahepatic bile ducts with the maximum diameter of 30 mm in CTV. The liver was normal in size with smooth capsules and an unevenly distributed rough echo. There are multiple well-defined hypoechoic mass lesions in the liver and bilateral kidneys. The comet's tail echoes diffused in the kidney ultrasound suggesting multiple calcifications. The spleen was not shown due to splenectomy. Three-dimensional reconstruction of the portal vein CTV-enhanced scan of P6 (e). The maximum diameter of the portal venous trunk was 19 mm (arrow). The spleen was markedly enlarged (asterisk), and intrahepatic portal vein branches were dilated, including the superior mesenteric vein (arrow), splenic vein (arrow), esophageal and gastric veins, and umbilical vein.

Multiple radiologic examinations revealed various degrees of portal hypertension in all of the cases (Supplementary Table S1). Both children and adults had enlarged spleens in ultrasound or computed tomography (CT) imaging. In the three pediatric cases with hematemesis (P2, P3, and P4), endoscopy or angiography was used to verify esophageal and gastric variceal bleeding (EGVB). Patient 3 and Patient 5 received splenectomy owing to severe hypersplenism and EGVB previously. The dilation of multiple portal collateral circulations in three cases (P2, P4, and P6) was revealed by CT venography (CTV) of portal veins (Figure 1). The liver morphology illustrated by imaging was diverse. Diffuse changes were displayed by heterogeneous internal echoes of ultrasonography in all cases. Most imaging reports included the diagnosis of “cirrhosis.” Nodular changes and irregular contours of the liver were shown in Patient 3 and Patient 4. Also, elevated fibroscan values in Patient 5 (51.5 kPa) and Patient 6 (11.9 kPa) ranged from fibrosis to “cirrhosis.” Three patients (P3, P5, and P7) had congenital dilation of the intrahepatic bile ducts manifesting as multiple cysts in the ultrasound and as high signal in the magnetic resonance cholangiopancreatography (MRCP), in which Patient 3 and Patient 5 also had polycystic kidneys (Figure 1 and Supplementary Figure S1).

### 3.6. Pathology

**Figure 2:**
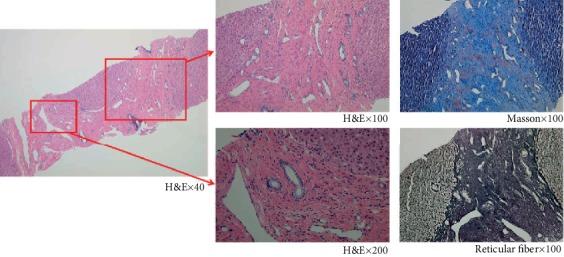
The pathological characteristics of a transcutaneous needle liver biopsy from P6. The H&E staining showed a disordered but intact lobular structure with periportal fibrosis and cystic or regular bile ducts lined with cuboidal epithelial cells in the portal area. There was no collapse of the fibrous scaffold in the reticular and Masson stainings which indicated the presence of collagen fibers in the fibrotic area.

All liver specimens from the three pediatric cases after liver transplantation (P1, P2, and P3) showed gray-white or gray-yellow nodules of different sizes on the liver's surface. The whole liver from Patient 1 shrunk and was divided into small nodules by forking fibers. There was a light-green 4 × 3 × 3 cm multilocular cyst in the liver of Patient 3, corresponding to congenital bile duct dilatation in the images. In the pathological sections of all cases, we observed thick bridging fibrous septa across hepatocyte cell islands but the lobular structures were still intact. There was no collapse of the fibrous framework in the reticular fiber staining. Masson staining showed extensive fibrosis of the liver tissue composed of fibrous septa with different widths (Figure 2). The cells in the portal area stained positive for CD7 and CD19, suggesting hyperplasic small bile ducts with irregular cavities.

### 3.7. Genetic Testing

We examined specific genes in two cases based on clinical features. The genetic and pathological testing of Patient 4 suggested CHF with a positive genotype for PFIC3, namely, *ABCB4* mutation-mediated MDR3 deficiency, which was histologically characterized by bile duct proliferation, portal inflammation, and fibrosis. Nevertheless, the pathological features of the liver, in this case, coincided with CHF. Also, electron microscopy imaging revealed cholestasis, broadening of partial liver cells, and bile capillaries which was consistent with elevated total bile acid (TBA) and *γ*-glutamyl transpeptidase (GGT) and cholestatic pruritus in PFIC. According to the decreased renal function and renal anemia due to polycystic kidney disease in Patient 5, we examined the *PKHD1* gene related to ARPKD but no mutation was found.

### 3.8. Treatment and Prognosis

Patients 1-4 were all candidates for liver transplantation. There was one child and one adult who received a splenectomy due to severe hypersplenism and upper gastrointestinal bleeding (P3, P5). In the group of four adults with mild symptoms and slow disease progression, the managements were mainly supportive treatment and follow-up. Oral *β*-blockers, such as propranolol, sustained the exacerbation of portal hypertension. Long-term use of liver protectors (e.g., glutathione and ursodeoxycholic acid) was beneficial for impaired liver function.

### 3.9. Analysis of CHF Cases in the Past 20 Years

From Chinese databases, we enrolled 63 cases in the past 20 years in China, including 30 children (10.18 ± 4.79 years old) and 33 adults (31.21 ± 14.31 years old); 88.89% (56/63) of whom were definitively pathologically diagnosed, and the others were clinically diagnosed according to their clinical manifestations and family histories [5–37]. Among them, CHF was reported in siblings aged from 6 to 21 years old in four families. The onset age, disease progression, and associated comorbidities were similar in the same family. In addition, family history of cirrhosis and polycystic liver and kidneys was evident in five sporadic adult patients. Moreover, only patients in five case reports took genetic testing. Heterozygous mutation of *PKHD1* and *PKD1* was detected in two adults with polycystic kidneys. One 18-year-old female, presenting with polycystic ovary syndrome and polycystic liver disease, was found to carry *NPHP2* and *CC2D2A* mutations [10]. Among two families who received high-throughput sequencing, *PKHD1* and *PKD1* mutations were detected, respectively, and Sanger sequencing confirmed that compound heterozygous mutations were from their parents [31, 37]. The proportion of liver hepatomegaly in children was significantly higher than that in adults (57% *vs*. 21%, *p* = 0.004, Table 2). There were no significant differences in any other manifestation between the two populations. Up to 50% of adult patients accompanied with malformation of kidneys and bile duct abnormalities. Besides, we found multiorgan involvement including the eyes, lungs, genital system, and central nervous system, which prompted us to evaluate the probability of CHF.

## 4. Discussion

CHF is an autosomal recessive inherited disease. It can manifest with hepatic fibrosis alone, or it can be an important component of various fibrocystic diseases involving multiple systems (Supplementary Table S2) [38–43]. Also, CHF is observed in X-linked and autosomal dominant inherited disease like oral-facial-digital syndrome type 1 and autosomal dominant polycystic kidney disease (ADPKD), respectively, and is reported in Prader-Willi syndrome, abernethy malformation, and different types of hepatic nodules (e.g., hepatocellular adenoma and hepatoblastoma) in individual case reports [44–47]. In our cases, we found that CHF could coexist with CBA and *ABCB4* mutation, but their association with CHF has not been verified. Also, image analysis suggested Caroli syndrome or polycystic kidney disease despite the lack of genetic testing, which was often categorized into the “hepatorenal fibrocystic disease” (HRFD) family [4].

Based on onset of symptoms, it is generally accepted that there are four clinical forms of CHF: portal hypertensive, cholangitic, mixed, and latent [48]. A considerable portion of adulthood-onset patients has latent disease progression that requires clinicians' attention. As the case review suggested, children with CHF had a more severe phenotype including a higher Child-Turcotte-Pugh (CTP) score and more severe complications of portal hypertension, and should be considered for isolated liver or combined liver-kidney transplantation early [49]. For adult patients, symptoms were diverse, such as fever and fatigue, unexplained liver injuries, or serendipitous polycystic kidney disease, compared with typical symptoms of portal hypertension in children. In a review of adult cases, clinical manifestations varied from asymptomatic to liver decompensation requiring transplantation. Adult patients could experience a period of agnogenic “cirrhosis” or other related syndromes [50]. Therefore, it is significant to reevaluate medical history and liver biopsy specimens before the definitive diagnosis. Image-based evaluation should be performed early to direct the clinical practice. The combination of high-resolution ultrasound and MRCP can noninvasively provide detailed information regarding the extent of kidney and hepatobiliary involvement [51]. Patients with CHF typically have the following imaging features: dysmorphic liver (usually hypertrophic left segment and atrophic right lobe), portal hypertension, and other DPMs (e.g., biliary hamartoma and Caroli syndrome) [52]. Moreover, a systemic radiological examination is necessary to assess whether other organs are involved, particularly for neuromuscular or renal abnormalities [53].

As in the cases mentioned above, one or more liver diseases could coexist with CHF such as CBA, CHB, and ICP. In particular, adult patients are more likely to develop other chronic liver disorders. These comorbidities could confuse clinical judgment and act as aggravating factors. When CHF is the principle cause of disease, it is necessary and challenging to distinguish the fibrotic change of histology due to other liver disorders. In summary, a detailed inquiry of family history, searching for extrahepatic evidence, and liver biopsy are pivotal for making a definitive and differential diagnosis. If there is an unexplained liver injury in adults, liver biopsy should be performed as early as possible. A second biopsy and evaluation is helpful in cases of unrelieved symptoms and persistent progression of the illness after treatment.

There is still a lack of sequencing panels and gene database covering CHF-related genes. In our study, the clinical features were inconsistent with gene mutations. For example, Patient 5 was clinically associated with typical polycystic kidney disease with renal insufficiency; however, no mutations were found in the *PKHD1* gene. Similarly, kidney and liver diseases were independent of each other and variability in severity couldn't be not explained by the type of *PKHD1* mutation in an ARPKD cohort [54]. As another example, an *ABCB4* mutation was detected but typical cholestasis was lacking in liver pathology of Patient 3. The association between portal hypertension in CHF and cholestasis in PFIC3 has not been reported and requires further investigation. Also, a case report found *TMEM67* mutations in an isolated CHF case instead of related syndromes [39]. Therefore, genetic tools play a limited role in the diagnosis of CHF and should be the focus of future studies.

Different principles of treatment are required based on illness severity at different ages. Prompt liver transplantation is necessary for severe portal hypertension and liver decompensation. For adult patients with occult onset, the current clinical work is more focused on timely diagnosis by liver biopsy, comprehensive assessment of multiple systems, and long-term surveillance of liver and kidney function, portal hypertension, and hepatocellular carcinoma. The management of CHF is directed toward supportive treatment and relieving complications including antifibrotic drugs, antibiotics for cholangitis, and surgical intervention of portal hypertension [55]. Familiar treatments of portal hypertension are embolization and endoscopic ligation for EGVB; splenectomy combined with portosystemic shunting is a better choice for treating repeated variceal bleeding [56, 57]. There is currently no drug that can effectively reverse liver fibrosis in clinical trials including colchicine, *γ*-interferon, angiotensin II receptor blockers, pirfenidone, and ursodeoxycholic acid [39]. Gene therapy or stem cell transplantation is not yet available but remains a promising direction. It is necessary to establish a clinical and genetic database for precise molecular diagnosis and a better understanding of disease pathways, which is the cornerstone of therapeutic breakthroughs [58].

## 5. Conclusions

In conclusion, CHF in children was characterized by severe complications of portal hypertension requiring liver transplantation, while adults often had atypical complaints upon onset. Therefore, nonpediatric clinicians should not ignore this rare kind of congenital liver disorder when they encounter unexplained portal hypertension or isolated injured liver function. If available, it is essential to perform a liver biopsy for early diagnosis. Adults with CHF vary a lot in clinical manifestation. Extrahepatic organ involvement and unusual progression of liver disease can provide clues of diagnosis. A multidisciplinary cooperation of hepatologists, radiologists, and pathologists is important to make a correct diagnosis. A systematic evaluation is favorable to diagnose CHF-associated syndromes and necessary to manage the disease thoroughly, including but not limited to renal, ophthalmic, pulmonary, genital, and neuromuscular involvement.

## Figures and Tables

**Table 1:** Basic clinical information of 8 cases with different onset ages.

No.	Sex	Age	Chief complaint	Course	Principal diagnosis	Hb (g/L)	WBC (10^9^/L)	PLT (10^9^/L)	ALT (U/L)	AST (U/L)	ALP (U/L)	GGT (U/L)	TBil (mmol/L)	INR	Alb (g/L)
P1	M	1 yr	Hyperpyrexia	1 month	CHF^§^ CBA Cholangitis after Kasai operation	110^∗^	9.13	205	51	68	553	265	6.2	1.04	37

P2	F	4 yr	Hematemesis	1.5 month	CHF^§^	86	3.71	189	268	230	813	500	12.3	0.94	39

P3	M	15 yr	Hematemesis and melena	8 years	CHF^§^ Caroli disease	52	5.63	649	17	19	129	26	3.7	1.15	27

P4	F	9 yr	Melena and bloating	2 months	CHF^§^ PFIC3	68^∗^	2.25	226	317.9	274.1	382	233	14.7	0.87	28.8

P5	F	26 yr	Bloating, edema, and oliguria	7 years	CHF CHB Intrahepatic biliary duct cystic dilation Polycystic kidneys^§§^	83^∗∗^	7.04	329	15	20	91	22	12	1.08	37

P6	F	26 yr	Isolated abnormal liver function	6 years	CHF	134	4.81	66	67	51	156	177	46.1	1.12	46

P7	M	26 yr	Isolated abnormal liver function	9 months	CHF Intrahepatic biliary duct cystic dilation	147	4.19	75	20	21	45	64	17.5	1.20	45

P8	F	60 yr	Abnormal liver function and edema	4 years	CHF	136	3.82	72	46	55	139	24	31.8	1.19	32

Abbreviations and normal range of each lab index in parentheses: P = patient; M = male; F = female; yr = years old; CHF = congenital hepatic fibrosis; CHB = chronic hepatitis B; CBA = congenital biliary atresia; PFIC3 = progressive familial intrahepatic cholestasis-type 3; Hb = hemoglobin (male: 130-175 g/L, female: 115-150 g/L); WBC = white blood cell (3.75 × 10^9^/L-9.5 × 10^9^/L); PLT = platelet (125 × 10^9^/L-350 × 10^9^/L); ALT = alanine aminotransferase (male: 9-50 U/L, female: 7-40 U/L); AST = aspartate aminotransferase (male: 15-40 U/L, female: 13-35 U/L); ALP = alkaline phosphatase (1~4 yrs: <281 U/L; 5~6 yrs: <269 U/L; 7~12 yrs: <300 U/L; male 13~17 yrs: <390 U/L; male 18~19 yrs: 40-129 U/L; male ≥ 20 yrs: 45-125 U/L; female 13~17 yrs: <187 U/L; female 18~19 yrs: 35-104 U/L; female 20~49 yrs: 35-100 U/L; female 50~79 yrs: 35-100 U/L; and female ≥ 80 yrs: 50-135 U/L); GGT = *γ*-glutamyl transpeptidase (male: 10-60 U/L, female: 7-45 U/L); TBil = total bilirubin (3.4-20.4 *μ*mol/L); INR = international normalized ratio (0.88-1.12); Alb = albumin (40-55 g/L). ^§^CHF: combined with liver decompensation; ^§§^polycystic kidneys combined with chronic renal dysfunction; ^∗^IDA: iron deficiency anemia; ^∗∗^RA: renal anemia.

**Table 2:** Difference analysis in clinical features between children and adults in 63 Chinese case reports.

Clinical features	Children (*N* = 30) *n* (%)	Adults (*N* = 33) *n* (%)	*p* value
Abnormal liver function	5 (17%)	9 (27%)	0.312
Liver transplant	4 (13%)	4 (12%)	1.00
Partial hepatectomy	5 (17%)	6 (18%)	0.874
Hepatomegaly	17 (57%)	7 (21%)	0.004
Splenomegaly	21 (70%)	22 (67%)	0.777
Splenectomy	3 (10%)	8 (24%)	0.137
EGVB	15 (50%)	19 (58%)	0.547
Ascites	5 (17%)	10 (30%)	0.204
Polycystic kidneys	8 (27%)	16 (48%)	0.075
Medullary sponge kidneys	4 (13%)	3 (9%)	0.700
Bile duct abnormalities	13 (43%)	19 (58%)	0.259
Portal vein malformations	3 (10%)	3 (9%)	1.000
Other systems	4 (13%)^†^	7 (21%)^††^	0.411

EGVB = esophageal and gastric variceal bleeding. ^†^Four cases including pulmonary interstitial fibrosis, uterine and kidney malformation, patent ductus arteriosus, and arachnoid cyst. ^††^Seven cases including arachnoid cyst and cerebral dysplasia, cerebral aneurysm, polycystic ovary syndrome, breast dysplasia, duodenal diverticulum tumor, macular degeneration, and IgG4-related autoimmune pancreatitis.

## Data Availability

All relevant data supporting the conclusions of this case report are displayed within this manuscript. More detailed data is available from the corresponding author upon request.

## Supplementary Materials

Supplementary Materials Supplementary Table S1: complications involved in portal hypertension and other imaging findings. Supplementary Table S2: autosomal recessive inherited syndromes related with CHF. Supplementary Figure S1: magnetic resonance cholangiopancreatography (MRCP) of P7 showed cystic and columnar expansion of intrahepatic bile ducts indicating a probability of congenital dysplasia (coronal planes a–c, sagittal planes d–f).
