# Supplementary material for: Congenital Hepatic Fibrosis in Children and Adults: Clinical Manifestations, Management, and Outcome—Case Series and Literature Review
Source: Gastroenterol Res Pract. 2020 Apr 21;2020:8284274. doi: 10.1155/2020/8284274 (PMC7191434; doi:10.1155/2020/8284274)
Supplement: Supplementary Materials — Supplementary Table S1: complications involved in portal hypertension and other imaging findings. Supplementary Table S2: autosomal recessive inherited syndromes related with CHF. Supplementary Figure S1: magnetic resonance cholangiopancreatography (MRCP) of P7 showed cystic and columnar expansion of intrahepatic bile ducts indicating a probability of congenital dysplasia (coronal planes a–c, sagittal planes d–f). [file 8284274.f1.pdf]

Supplementary Materials

Supplementary Table S1 Complications involved in portal hypertension and other imaging findings

No.	EGVB	hepatomegaly	splenomegaly	lymphadenectasis	Serous cavity effusion
P1		+	+	+	
P2	+	+	+		
P3	+		+		+（ascites）
P4	+		+	+	+(polyserositis)
P5			+		
P6			+		
P7			+		
P8			+		

Supplementary Table S2 Autosomal recessive inherited syndromes related with CHF

CHF associated AR syndrome	Clinical features	Mutated genes
Caroli's syndrome	Caroli's disease, congenital hepatic fibrosis	*PKHD1*
ARPKD	Renal cysts, hepatic fibrosis, pulmonary hypoplasia, systemic hypertension	*PKHD1*
Nephronophthisis(NPHP)	cerebello-ocular-renal syndromes, liver fibrosis, skeletal defects, scoliosis, cleft palate, situs inversus	*NPHP1*, *INVS*, *NPHP3*, *NPHP4*, *IQCB1*, *CEP290*, *GLIS2*, *RPGRIP1L* ,*NEK8* ,*TMEM67* ,*TTC21B* ,*WDR19* ,*ZNF423* ,*CEP164* ,*ANKS6*
Meckel syndrome(MKS)	renal cystic disease, occipital encephalocele, polydactyly, fibrotic/cystic changes of the liver, orofacial clefts	*MKS1*, *TMEM216*, *TMEM67*, *CEP290*, *RPGRIP1L*, *CC2D2A*, *NPHP3*, *TCTN2*, *B9D1*, *B9D2*, *TMEM231*, *KIF14*
Joubert syndrome and related disoeders(JSRD, e.g., COACH, Arima syndrome)	Cerebellar vermis hypoplasia(molar tooth sign, MTS), episodic tachypnea or apnea, ocular motor apraxia, retinitis pigmentosa, ataxia, oligophrenia, renal abnormalities, coloboma, psychomotor retardation	*INPP5E*, *TMEM216*, *AHI1*, *NPHP1*, *CEP290*, *TMEM67*, *RPGRIP1L*, *ARL13B*, *CC2D2A*, *OFD1*, *TTC21B*, *KIF7*, *TCTN1*, *TMEM237*, *CEP41*, *TMEM138*, *C5orf42*, *TCTN3*, *ZNF423*, *TMEM231*, *TCTN2*
Bardet–Biedl syndrome(BBS)	retinitis pigmentosa, polydactyly, mental retardation, hypogonadism, renal dysfunction	*BBS1*, *BBS2*, *ARL6*, *BBS4*, *BBS5*, *MKKS*, *BBS7*, *TTC8*, *BBS9*, *BBS10*, *TRIM32*, *BBS12*, *MKS1*, *CEP290*
Jeune asphyxiating thoracic dystrophy (Jeune Syndrome, JATD)	Small/narrow chest, shortened bones of the arms and legs, cone shaped epiphyses, renal dysfunction, liver disease	*IFT80*, *IFT140*
Cranioectodermal dysplasia	bone abnormalities, dysplasia of ectodermal tissues, nephronophthisis	*WDR35*, *IFT122*, *WDR19*, *IFT43*
Ellis-van Creveld syndrome	abnormal bone growth, congenital heart disease, postaxial polydactyly, dystrophic nails and teeth, retinal degeneration.	*EVC*, *EVC2*
Renal-hepatic-pancreatic dysplasia(RHPD)	renal dysplasia, pancreatic fibrosis, hepatic dysgenesis	*NPHP3*

Ivemark syndrome	asplenia, heart malformations, abnormal lung lobation, localized renal dysplasia	*unidentified*

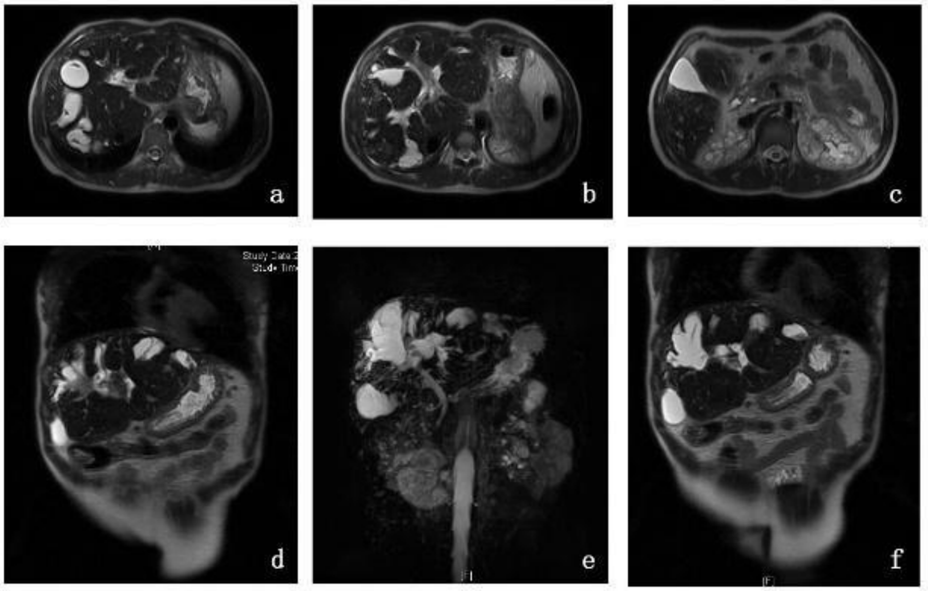

Supplementary Figure S1: Magnetic Resonance Cholangiopancreatography (MRCP) of P7 showed cystic and columnar expansion of intrahepatic bile ducts indicating a probability of congenital dysplasia (coronal plane a~c, sagittal plane d~f).
